# Distortions of the Spinal Column

**Published:** 1847-09

**Authors:** 


*Distortions of the Spinal Column.*—A work by Mr. James Coles on this subject was published about two years since, and although the principle of treatment inculcated has been for some time before the profession, and has not been generally adopted, its advantages are insisted upon anew, and ably supported by facts and reasoning, and the interests of our readers, we think, will be consulted by making it a subject for this Report. Mr. Coles strongly insists that the cause of spinal curvature, as a primary disease, is constitutional debility, most frequently, if not always, of strumous origin, and that local debility is but a secondary cause. He denies that local influences can cause the disease when constitutional predisposition does not exist, and accordingly he attributes much less influence to stays, improper attitudes, want of exercise, schools, dress, and the habits of society, than do other surgeons; believing that, at most, they can only produce secondary effects. Attention is, however, drawn to the important distinction between primary and secondary distortion of the spinal column, the latter being produced by the local causes above referred to, and great mischief having arisen in practice from a want of this discrimination, inasmuch as the constitutional disease has been frequently aggravated by remedial measures applicable only to that which has a local origin.

All the cases of spinal distortion are referred to four varieties, the lateral, the anterior and the posterior curvatures, and the angular projection; and the circumstances which more especially facilitate the progress of the disease are two, the superincumbent weight of the body on the spine, and the fatigue and exhaustion occasioned by maintaing an erect position. The indications of treatment are three: 1st. To relieve the distorted spine of its superincumbent weight. 2d. To remove local active disease or local debility. 3d. To restore the general health.

Mr. Coles demonstrates the impracticability of fulfilling the first indication by any artificial contrivance whilst the body is in an erect position; it can only be effected by the recumbent position. This position may be supine, prone, or on either side, but there are insuperable objections to this supine and lateral positions. The inclined plane, so much adopted of late years, the patient lying on the back, is erroneous in principle as a remedy, and is attended with innumerable evils. The "facial-horizontal position," on a soft medium, as a feather-bed, appears to have been first recommended in a published essay by Mr. Bampfield, but the position had been previously adopted in practice by the late Dr. Verral, who invented the 'prone couch." This couch, in its simplest form, consists of a board having an angle corresponding with the bend of the hips; it is placed on a sofa. It must be carefully fitted to the patient's size, and admit of easy variations in its angles.

The objections to the supine position are manifold and serious: 1. The injurious effects of pressure, not only upon the integuments covering the sacrum, trochanters, and other projecting bones, but also upon the bones themselves. The continued pressure upon the muscles also cannot fail to interfere very seriously with their restoration to health and power; nay, it may even lead to a very considerable absorption of their substance, and, as the flexors of the body are naturally more powerful than the extensors, much mischief may be caused by the slightest lesion of this sort; a confirmed stoop is a very frequent result of the prolonged use of the reclining-board. 2. The continued pressure of the thoracic vicera upon the spinal and sympathetic nerves, and upon the great blood-vessels that lie upon the anterior surface of the vertebral column, can scarcely fail to produce a certain amount of disturbance in the functions of innervation and circulation. Hence, probably, the headache, difficulty of breathing, palpitations of the heart, indigestion, obstinate constipation, weakness of the bladder, hemorrhoids, varicose state of the veins, and œdema of the legs, &c., which are not unfrequently observed in patients who have been long confined to the horizontal supine position. Besides these and similar ailments, such invalids often experience a marked impairment of vision, in consequence of the manner in which the rays of light fall upon the eye, and of their reflection from the white ceiling of the room, upon which the eye must necessarily rest during the greatest part of the time spent upon the plane.

The main object of Mr. Coles' work is to illustrate and establish the fficacy of the prone position. Its advantages are, that the muscles of the spine are not obstructed in their action, nor is their structure absorbed by pressure; that all the injurious effects produced by internal pressure of the viscera on the diseased spine and the adjacent parts are avoided; that excoriations and sloughings do not occur; the anterior surface of the body, from its yielding nature, bearing the weight of the frame without injury or inconvenience; that there is easy access to the diseased parts, and local remedies for active disease or local debility can be applied; that patients can maintain this position by night and by day with comfort to the feelings, improved appetite, and regularity of bowels; that whilst the patient resposes upon the horizontal or slightly sloping portion of the plane, which extends from the point of the shoulder to the bend of the hips, the lower extremities are bent at an angle more or less acute, according to the nature of the case; and by being thus suspended upon the sloping position of the couch, are made to keep up a steady and constant *extension* by their weight upon the distorted or diseased parts; the effect of this gentle and continued extension materially assisting in straightening the back, and where caries of the bones and ulceration of the cartilages exist, separating the diseased surfaces and counteracting the pressure, which even the contraction of the muscles occasions and renders injurious. The position thus removes a principal cause of the absorption of bone, inflammatory action, increase of deformity, paralysis, and other symptoms. In a quotation from Mr. Liston, it is remarked that the prone position may be most favorable in certain diseases, by taking the pressure off the diseased parts, by preventing the carious bodies of the vertebræ from falling in upon one another, and by assisting the return of the blood from the numerous veins contained in the bodies of the vertebræ and in the spinal canal.

Mr. Coles admits the propriety of the usual mode of practice in lateral curvature, so long as it is *secondary* in its nature, by which he means dependent upon local causes, but the moment marked symptoms of constitutional debility become apparent, which, in nine cases out of ten, is antecedent to or coeval with the first signs of distortion, all the exercises generally employed to produce muscular strength are injurious, and the prone position should be immediately adopted.

The prone position, according to Mr. Coles, is applicable to confirmed cases of lateral curvature, and most essential when inflammation or disease of the bone exists; to both mild and severe cases of posterior projection; to injuries of the spine and paralysis of the lower extremities, whether caused by accident or otherwise; to all cases of psoas abscess, both in the early stages and when matter is pointing, or has made its way anteriorly; to the incipient stage of disease of the vertebral column; and to inflammation and injuries of the spinal marrow, and of its investing membranes; to deformities of the chest, and to disease of the hip-joint. Mr. Coles states that in many of these cases, otherwise incurable, the prone position will enable us to affect a cure, and that when a cure is impossible, it affords certain relief and a great chance of improvement.

For the lateral depression of the chest and prominent sternum, Dupuytren had recommended frequent pressure upon the sternum. Mr. Coles recommends the prone couch and the pressure which results from the weight of the chest upon the mattress, which can be increased, diminished, or removed, by altering the angle at which the body rests, and by rendering the material of the mattress either hard or soft. Pressure thus produced does not interfere with the elasticity of the chest, and may be sustained for a long time without inconvenience.

To produce extension without the aid of merely mechanical means, which Mr. Coles condemns, a special contrivance is had recourse to, and when the curve is situated high up in the vertebral column, or in the neck, extension may be effected by the use of a head-swing. This modified extension, combined with the relaxation of all the parts having any influence upon the hip-joint, tends to separate the diseased surfaces in hip-joint disease, and renders the prone position, in Mr. Coles' estimation, almost a specific in its treatment. Thus the prone position for the purpose, with other indications, of taking off the superincumbent weight of the head and of the viscera, according to Mr. Coles, should be our first principle of treatment in spinal affections; but where there is local disease present or suspected, he resorts to the measures recommended by other surgeons, sometimes employing counter-irritation, issues, blisters, and especially local applications of iodine, and in the main preferring mild to severe measures. For the constitutional debility he recommends great attention to the digestive organs and the secretions, a generous diet, with the moderate use of porter rather than of wine, iodine and steel, as exercising the most direct medicinal influence, and he attaches much importance on administering the latter in small but repeated doses.

For the purpose of rectifying the deviation when no pain or tenderness is present, he employs stretching by the muscular efforts of the patient, repeated about every second day; manipulation and pressure, which should be directed upon correct anatomical and physiological principles; friction, which is a most important means of stimulating the activity of venous circulation, and of promoting absorption; and local muscular exercise, which excites the arterial circulation, propels blood to the parts, and, promoting deposition, increases the power and activity of the muscles. These measures may be made to produce the best effects, but if injudiciously employed, are calculated to do the greatest possible mischief.

The following illustrative cases are condensed from Mr. Coles' essay :—

A boy, twelve years of age, affected with a posterior projection, the disease affecting several of the lower dorsal and lumbar vertebræ, was greatly improved by maintaining the prone position for five months, and perfectly restored at the end of seven months. A boy, three years and a half old, with a posterior projection, including only three bones, the middle one evidently diseased, was completely cured by remaining on the prone couch eight months. A boy, seven years and a half old, of an intensely scrofulous habit, with very considerable angular projection, including eight dorsal vertebræ, and terminating in a sharp point, over which the skin was stretched, pale, and shining, having been placed under treatment for twelve months, during which there was every symptom of caries of the vertebræ, the prone position being steadily maintained, on his discharge was able to walk upright, was in good general health, the projection of the spine much reduced, and the carious state cured ; the only remaining deformity being the result of anchylosis of the diseased bones. A boy, twelve years of age, with a considerable posterior projection, of an angular kind, in the neck and upper dorsal vertebræ, the ribs on each side flattened, the lower end of the sternum projected outwards, suffering also from dyspnœa and violent palpitation and cough, the body greatly emaciated, the lower limbs completely paralysed, and all control over the evacuations lost, experienced immediate relief from being placed on a prone couch ; and having been kept in that position for eighteen or twenty months, with the aid of constitutional and local treatment for various symptoms which presented themselves, sensation and the power of motion gradually returned in the limbs, with the natural command over the bowels and bladder ; pain and heat in the spinal column subsided, and the projection diminished ; the deformity of the chest also subsided, and with it the pectoral and cardiac symptoms ; the boy was ultimately entirely cured, with very little deformity remaining to indicate the original seat of the disease. A young lady, with a very considerable lateral curvature, and a counter curve in the loins, placed on the "prone couch," both by night and by day, uniformly and rapidly improved, and was ultimately quite cured. A policeman, from an injury received on duty, had paralysis of the side, and the spine in the lumbar region projected, and presented an angular appearance, with great pain on pressure and motion, and paralysis of the bladder ; this case was completely cured. Other cases of a most unpromising character are recited, in which success was complete ; and Mr. Coles most strongly insists that the prone position is also applicable with the greatest possible advantages to the treatment of hip-joint disease.—*Report on Surgery by Henry Ancell, Esq.—Ibid.*

